# Helps to Gain Patience

**Published:** 1890-05-17

**Authors:** 


### WORDS OF CONSOLATION.

### HELPS TO GAIN PATIENCE.

We have settled in our minds that natural patience, or in other words a placid disposition born in a person, is of small use in a long illness. It helps us on a little way, but sooner or later it fails us when we are most in need of it, and we find, to our intense mortification, that we are no better than our neighbours. Indeed it is well that it should be so, for if we thought our patience was truly our own, the offspring of our own hearts, we should become vain and forget that all good gifts, whether of mind or body, come from our Almighty Father.

There is no cure for impatience other than by giving up ourselves entirely to His keeping, by dwelling in Him, trusting in Him, and leaning on the Everlasting Arms, which He ever stretches out to support His beloved children. But there are helps to the cure; and by strict watchfulness over ourselves we may do much through Christ who strengthens us.

1st. When you feel impatient, if possible do not speak. If you do, it is most probable that you will say words which will cause you sorrow and grief afterwards. Many of us know the story of the woman who had a bad tempered husband. She heard that a quack doctor advertised a sure cure for such a grievance, so she hurried to him to gain the benefit for her own case. The man gave her a bottle full of a clear red liquid, telling her that as soon as her husband began to storm she was to fill her mouth with some of its contents and hold it there from three to five minutes. She carried away the mixture and at the end of a month came back for more saying the effect was marvellous, her husband was becoming quite good tempered and she wanted to cure him entirely. "Foolish woman," said the clever fellow, "cannot you see that the only secret of the cure is in holding your tongue." So true is it that silence is golden.

2nd. But should it be necessary to speak, then send up a short prayer to God for help, such as, Lord save me, Lord help me. Any like words will do, only commend yourself to God and then be strong in His Might and fear not.

3rd. Do not torment yourself by continually fancying you are impatient, it will only make you more so; and do not exaggerate your feelings even to yourself.

4th. If you have shown impatience to a friend or a nurse, distinctly say you know you did it, that you were wrong and are sorry for it and do not try to excuse yourself. It is a useful habit to acquire and will teach you humanity if practised aright. Besides it is the only restitution you can make to them, and will be a blessing to both, showing your feeling for the sin and that you do not fall into it without true repentance.

5th. Notice those persons and things which make you most impatient and try and avoid them if possible. If it is impossible, then specially watch those weak points and be on your guard at all times against them. This is another case where darting up a prayer to God to be with you and strengthen your heart is your safest help.

6th. Do not expect to be free from impatience altogether whilst you are weak, and remember that though sickness is an especial time for the exercise of patience yet it is that state in which your deepest nature is stirred up and you are aggravated by things which in health would not affect you.

